# N-terminal pro-brain natriuretic peptide and coronary collateral formation in patients undergoing primary percutaneous coronary intervention

**DOI:** 10.1007/s00380-021-01866-3

**Published:** 2021-05-28

**Authors:** Bo Eun Park, Jang Hoon Lee, Hyeon Jeong Kim, Hong Nyun Kim, Se Yong Jang, Myung Hwan Bae, Dong Heon Yang, Hun Sik Park, Yongkeun Cho, Shung Chull Chae

**Affiliations:** 1 Department of Internal Medicine, Kyungpook National University Hospital, 130, Dongdeok-ro, Jung-gu, Daegu, 41944 Republic of Korea; 2 School of Medicine, Kyungpook National University, Daegu, Republic of Korea

**Keywords:** Collateral circulation, N-terminal pro-B type natriuretic peptide, Acute myocardial infarction, Percutaneous coronary intervention

## Abstract

There is insufficient information on the relationship between the N-terminal pro-brain natriuretic peptide (NT-proBNP) level and collateral circulation (CC) formation after primary percutaneous coronary intervention (PCI) in patients with ST-segment elevation myocardial infarction. We analyzed 857 patients who underwent primary PCI. The serum NT-proBNP levels were measured on the day of admission, and the CC was scored according to Rentrop’s classification. Log-transformed NT-proBNP levels were significantly higher in patients with good CC compared to those with poor CC (6.13 ± 2.01 pg/mL versus 5.48 ± 1.97 pg/mL, *p* < 0.001). The optimum cutoff value of log NT-proBNP for predicting CC was 6.04 pg/mL. Log NT-proBNP ≥ 6.04 pg/mL (odds ratio 2.23; 95% confidence interval 1.51–3.30; *p* < 0.001) was an independent predictor of good CC. CC development was higher in patients with a pre-TIMI flow of 0 or 1 than those with a pre-TIMI flow of 2 or 3 (22.6% versus 8.8%, *p* = 0.001). The incidence of left ventricular (LV) dysfunction (< 50%) was greater in patients with a pre-TIMI flow of 0 or 1 (49.8% versus 35.5%, *p* < 0.001). The release of NT-proBNP was greater in patients with LV dysfunction (34.3% versus 15.6%, *p* < 0.001). The incidence of good CC was greater in patients with log NT-proBNP levels ≥ 6.04 pg/ml (16.8% versus 26.2%, *p* = 0.003). The association between NT-proBNP and collateral formation was not influenced by pre-TIMI flow and LV function. NT-proBNP appears to reflect the degree of collateral formation in the early phase of STEMI and might have a new role as a useful surrogate biomarker for collateral formation in patients undergoing primary PCI.

**Supplementary Information:**

The online version contains supplementary material available at 10.1007/s00380-021-01866-3.

## Introduction

The coronary collateral circulation (CC) is thought to provide blood for the ischemic myocardium and an interconnecting network to alleviate the burden of myocardial ischemia [1–3]. It is well known that well-developed CCs can reduce the size of infarct, preserve ventricular function, improve myocardial remodeling, prevent the formation of left ventricular (LV) aneurysm, resulting in fewer future cardiovascular events and improved rates of survival [4–7].

N-terminal pro-brain natriuretic peptide (NT-proBNP) is secreted from the myocardium due to increased myocardial wall stress [8]. Recently, it has been found that NT-proBNP can act as a stimulator of angiogenesis [9, 10]. However, in patients with ST-segment elevation myocardial infarction (STEMI), there insufficient information on the relationship between NT-proBNP and collateral formation after primary percutaneous coronary intervention (PCI). In the present study, we sought to elucidate the potential mechanism regarding association of the NT-proBNP level with coronary collateral formation in patients with STEMI after primary PCI.

## Materials and methods

This observational study included 857 consecutive patients who underwent primary PCI after STEMI and were enrolled in the Korean Acute Myocardial Infarction registry (KAMIR). The KAMIR is a Korean, prospective, open, observational, multicenter, online registry of AMI with support of the Korean Society of Cardiology that was initiated in November 2005; details of the KAMIR have been published previously [11]. The study was approved by the by the Institutional Review Board of Kyungpook National University Hospital (KNUIH 2011-11-023), and all patients provided written informed consent to participate. The study was performed in accordance with the ethical standards outlined in the 1964 Declaration of Helsinki and its later amendments or comparable ethical standards.

**Fig. 1:**
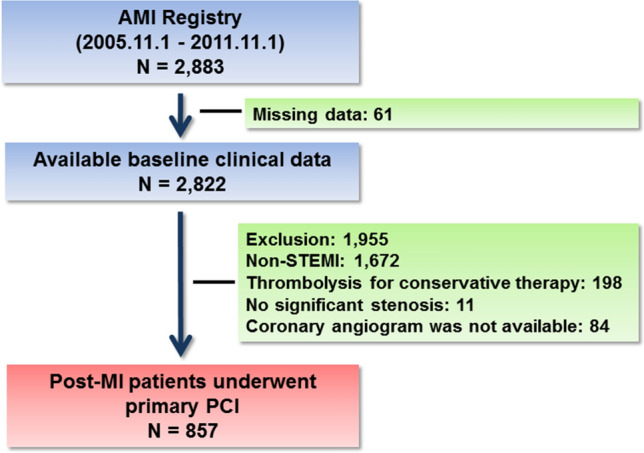
Flow diagram of the study subjects. *PCI* percutaneous coronary intervention, *STEMI* ST-segment elevation myocardial infarction

The flow diagram of the study is shown in Fig. 1. Between November 2005 and November 2011, 2883 patients with AMI were recruited. Baseline clinical data, including NT-proBNP, were available for 2822 patients; among whom, 1672 who had a non-STEMI, 198 who underwent thrombolysis or conservative treatment, 11 who had no significant stenosis, and 84 without available baseline angiogram were excluded.

AMI was diagnosed based on a characteristic clinical presentation, serial changes on electrocardiogram indicating infarction, and an increase in cardiac enzyme levels [12]. STEMI was defined by new ST elevation in two or more contiguous leads, measuring more than 0.2 mV in leads V1–V3, or 0.1 mV in all other leads. We analyzed the baseline clinical characteristics, angiographic findings, procedural data, and medications before hospitalization. The left ventricular ejection fraction (LVEF) was determined using two-dimensional echocardiography at the index hospitalization. NT-pro-BNP was measured at the time of admission using an electrochemiluminescence immunoassay method (Modular Analytics E170; Roche Diagnostics, Mannheim, Germany).

Coronary CC data were collected at baseline through standard angiography, with six views of the left coronary artery and two views of the right coronary artery and used for the interpretation of collateral vessels. CC was scored according to Rentrop’s classification [13]. A grade of 0 was given for no visible collaterals, 1 for a filled small side branch, 2 for filled major side branches of the main epicardial vessel, and 3 for main epicardial vessels filled by collaterals. Grades 0 and 1 were regarded as poor, and grades 2 and 3 were regarded as good CC. All data were entered into an electronic web-based case report form. The KAMIR protocol was approved by the ethics committee.

Data are shown as mean ± SD for continuous variables, and percentages for categorical variables. Student’s *t* test was used to perform all comparisons between the baseline variables and the continuous variables, and the Pearson Chi-square test was used to compare the categorical variables. Patients were categorized into two groups according to the presence of coronary CC: poor collateral circulation (*n* = 693) and good collateral circulation (*n* = 164). Univariate analyses were performed to determine the predictors for good CC. A logistic regression analysis model was used to compute the odds ratios (ORs) and 95% confidence intervals (CIs) of independent predictors of good collateral circulation. Variables with *p* values < 0.05 on univariate analysis were entered into the multivariate logistic regression model, namely the presence of preinfarction angina, pre-thrombolysis in MI (TIMI) flow grade of 0 or 1, multivessel disease, left anterior descending artery (LAD) as a culprit vessel, symptom-to-door time > 6 h, and log-transformed NT-proBNP. We estimated the receiver-operating characteristic (ROC) curves and the areas under the ROC curves (AUC) of NT-proBNP to identify the optimum cutoff value in the corresponding logistic models. The serum levels of NT-proBNP were compared within the collateral grade categories 0, 1, and 2/3, and with the analysis of variance according to the symptom-to-door time, presence of preinfarction angina, pre-TIMI flow of 0 or 1, and multivessel disease. For all analyses, a 2-sided *p* value < 0.05 was considered statistically significant. Statistical analysis was performed using SPSS software (version 18.0; SPSS Inc., Chicago, IL, USA).

## Results

**Fig. 2:**
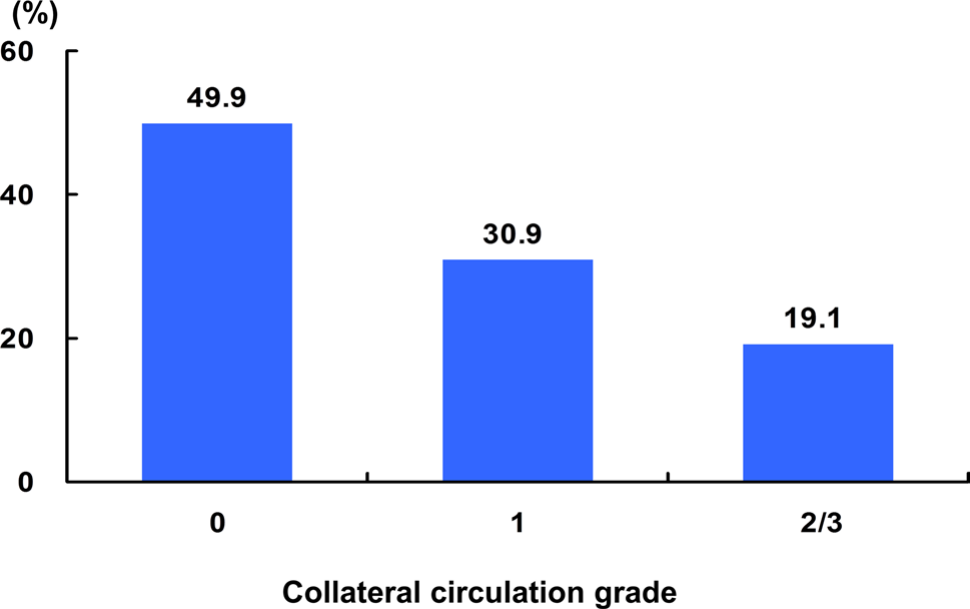
Distribution of coronary collateral circulation

**Fig. 3:**
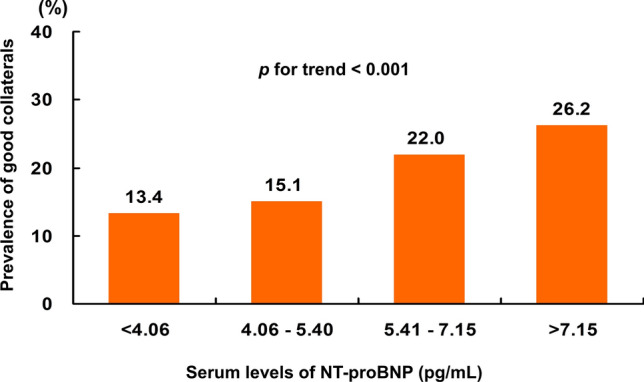
Prevalence of collateral circulation to the infarct-related artery based on a quartile of the serum N-terminal pro-B type natriuretic peptide levels

The mean age of the study population was 63 ± 12 years, and 634 (74.0%) were men. Overall, 429 (50.1%) had angiographic evidence of CC, including 265 (30.9%) with grade 1 and 164 (19.1%) with grade 2 or 3 (Fig. 2). The baseline characteristics of the study population are shown in Table 1. Patients with good CC had a greater prevalence of preinfarction angina (*p* = 0.038) and symptom-to-door time > 6 h (p = 0.009). Log-transformed NT-proBNP levels were significantly higher in patients with good CC compared to those with poor CC (6.13 ± 2.01 pg/mL versus 5.48 ± 1.97 pg/mL, *p* < 0.001). Among the angiographic findings, patients with good CC had a greater prevalence of multivessel disease (*p* = 0.022), pre-TIMI flow grade of 0 or 1 (*p* < 0.001), and a lower prevalence of LAD-related MI (*p* < 0.001) (Table 2). The prevalence of collaterals increased as the quartiles of serum NT-proBNP levels increased, from 13.4% in quartile 1 (< 4.06 pg/mL) to 15.1% in quartile 2 (4.06–5.40 pg/mL), 22.0% in quartile 3 (5.41–7.15 pg/mL), and 26.2% in quartile 4 (< 7.15 pg/mL) (*p* for trend) (Fig. 3).

**Table 1:** Clinical characteristics of study subjects

Variables	Overall (*N* = 857)	Collateral circulation		*p* value
		Poor (*N* = 693)	Good (*N* = 164)	
Demographics				
Age (year-old)	62.8 ± 12.2	62.9 ± 11.7	62.3 ± 12.9	0.585
Male	634 (74.0%)	516 (74.5%)	118 (72.0%)	0.510
Body mass index (kg/m^2^)	23.7 ± 3.1	23.7 ± 3.2	23.6 ± 3.0	0.538
Initial presentation				
Preinfarct angina	513 (59.9%)	403 (58.2%)	110 (67.1%)	0.038
Killip class > 1	222 (25.9%)	171 (24.7%)	51 (31.1%)	0.093
Symptom-to-door time > 6-h	217 (25.7%)	163 (23.8%)	54 (33.8%)	0.009
Left ventricular ejection fraction	49.6 ± 10.7	49.8 ± 10.6	48.5 ± 11.5	0.161
Past history				
Coronary heart disease	85 (9.9%)	62 (8.9%)	23 (14.0%)	0.050
Hypertension	601 (70.1%)	482 (69.6%)	119 (72.6%)	0.449
Diabetes mellitus	204 (24.0%)	162 (23.5%)	42 (25.8%)	0.550
Hyperlipidemia	188 (22.4%)	155 (22.9%)	33 (20.4%)	0.494
Current smoking	419 (48.9%)	340 (49.1%)	79 (48.2%)	0.825
Laboratory findings				
Hemoglobin (g/dL)	13.8 ± 1.8	13.9 ± 1.8	13.8 ± 1.8	0.797
Serum uric acid (mg/dL)	5.5 ± 1.8	5.5 ± 1.8	5.2 ± 1.7	0.110
eGFR (mL/min)	85.7 ± 26.2	84.2 ± 26.3	87.5 ± 24.6	0.136
Serum peak cTnI (ng/mL)	97.3 ± 181.6	94.0 ± 165.5	81.1 ± 97.5	0.335
Total cholesterol (mg/dL)	181.4 ± 44.0	181.6 ± 44.1	182.6 ± 45.7	0.805
Log NT-proBNP (pg/mL)	5.54 ± 2.14	5.48 ± 1.97	6.13 ± 2.01	< 0.001
Medications prior to admission				
Antiplatelet agent	60 (7.9%)	45 (7.2%)	15 (10.7%)	0.167
Beta-blockers	33 (4.3%)	23 (3.7%)	10 (7.2%)	0.067
ACE-Is/ARBs	48 (6.3%)	40 (6.4%)	8 (5.8%)	0.767
Statins	31 (4.1%)	22 (3.5%)	9 (6.5%)	0.115
Nitrates	19 (2.9%)	14 (2.6%)	5 (4.5%)	0.271

Data expressed as mean ± SD or number (percent)

*eGFR* estimated glomerular filtration rate, *cTnI* cardiac troponin I, *NT-proBNP* N-terminal pro-B type natriuretic peptide, *ACE-Is* angiotensin converting enzyme inhibitors, *ARBs* angiotensin type II receptor blockers

**Table 2:** Angiographic and procedural characteristics of study subjects

Variables	Overall (*N* = 857)	Collateral circulation		*p* value
		Poor (*N* = 693)	Good (*N* = 164)	
Coronary artery disease				0.067
Left main, isolated	2 (0.2%)	1 (0.1%)	1 (0.6%)	
Left main, complex	13 (1.5%)	12 (1.7%)	1 (0.6%)	
1-vessel disease	552 (64.4%)	459 (66.2%)	93 (56.7%)	
2-vessel disease	195 (22.8%)	152 (21.9%)	43 (26.2%)	
3-vessel disease	95 (11.1%)	69 (10.0%)	26 (15.9%)	
Multivessel disease	305 (35.6%)	234 (33.8%)	71 (43.3%)	0.022
Infarct related artery				< 0.001
Left main stem	15 (1.8%)	11 (1.6%)	4 (2.4%)	
Left anterior descending artery	417 (48.7%)	360 (51.9%)	57 (34.8%)	
Left circumflex artery	82 (9.6%)	69 (10.0%)	13 (7.9%)	
Right coronary artery	343 (40.0%)	253 (36.5%)	90 (54.9%)	
Pre-TIMI flow 0/1	642 (74.9%)	497 (71.7%)	145 (88.4%)	< 0.001
Post-TIMI flow 3	804 (96.2%)	652 (96.3%)	152 (95.6%)	0.820
Drug-eluting stent	568 (66.3%)	452 (65.2%)	116 (70.7%)	0.254

Data expressed as mean ± SD or number (percent)

*TIMI* thrombolysis in myocardial infarction

**Fig. 4:**
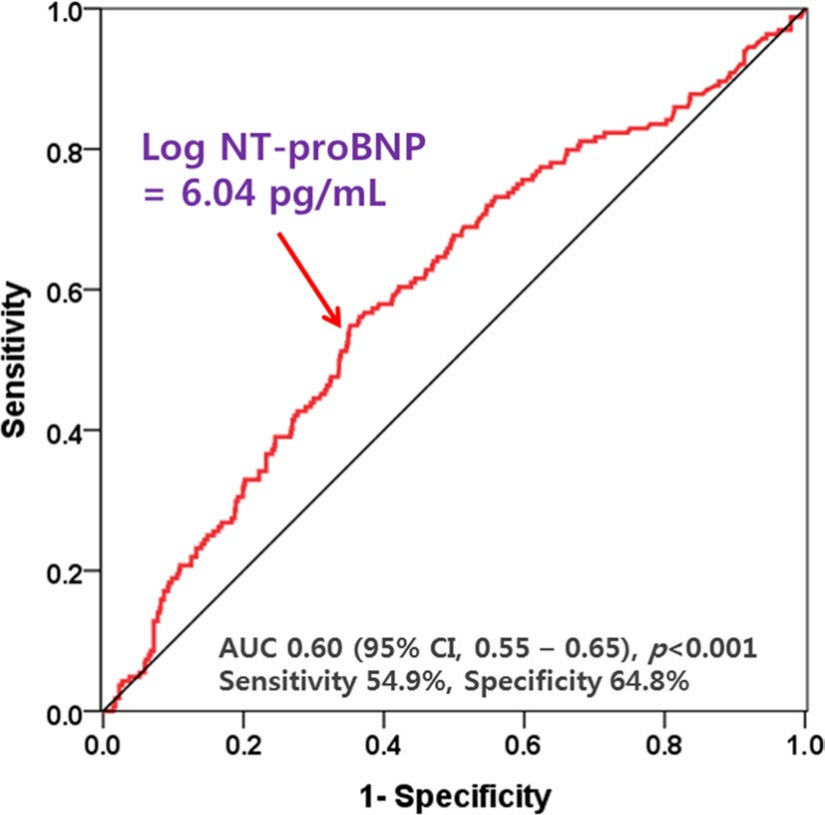
Receiver-operating characteristics analysis of N-terminal pro-B type natriuretic peptide for good collateral circulation. *AUC* area under the curve, *CI* confidence interval

In the ROC curve, the AUC of log NT-proBNP for predicting good CC was 0.60 (sensitivity, 54.9% and specificity, 64.8%), and the optimum cutoff value was 6.04 pg/mL (Fig. 4). In the multivariate logistic regression model, log-transformed NT-proBNP ≥ 6.04 pg/mL (OR 2.23; 95% CI 1.51–3.30; *p* < 0.001) was an independent predictor of good CC, in addition to a pre-TIMI flow grade of 0 or 1 (OR 2.89; 95% CI 1.71–4.90; *p* < 0.001) and LAD-related MI (OR 0.47; 95% CI 0.32–0.68; *p* < 0.001) was an independent predictor of good CC after adjusting for confounding variables (Table 3).

**Table 3:** Multivariate logistic regression model for good collateral circulation

	Odds ratio	95% confidence interval	*p* value
Preinfarction angina	1.22	0.83–1.78	0.316
Pre-TIMI flow 0 or 1	2.89	1.71–4.90	< 0.001
Multivessel disease	1.36	0.94–1.97	0.100
Left anterior descending artery culprit	0.47	0.32–0.68	< 0.001
Symptom-to-door time > 6-h	1.19	0.79–1.79	0.409
Log NT-proBNP ≥ 6.04 pg/mL	2.23	1.51–3.30	< 0.001

*TIMI* thrombolysis in myocardial infarction, *NT-proBNP* N-terminal pro-B type natriuretic peptide

**Fig. 5:**
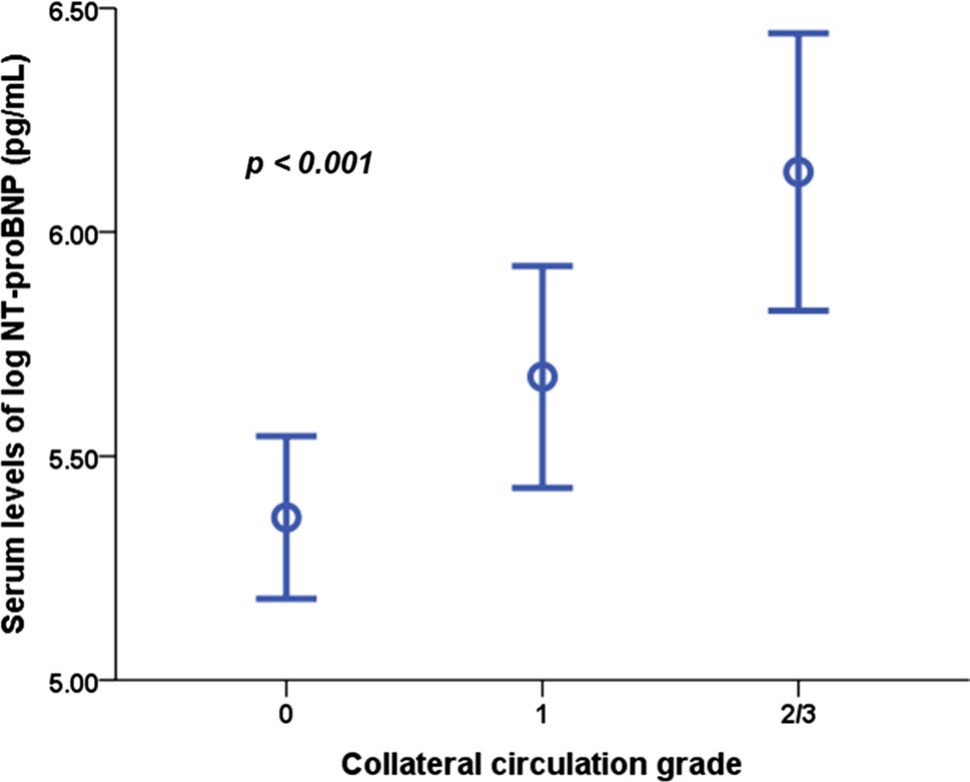
Serum levels of N-terminal pro-B type natriuretic peptide based on the grade of collateral circulation. *AUC* area under the curve, *CI* confidence interval

The serum levels of NT-proBNP were compared within the CC grade categories 0, 1, and 2/3. The serum levels of NT-proBNP increased with CC grade (5.36 ± 1.91 pg/mL versus 5.68 ± 2.04 pg/mL versus 6.13 ± 2.01 pg/mL, *p* < 0.001) (Fig. 5).

**Fig. 6:**
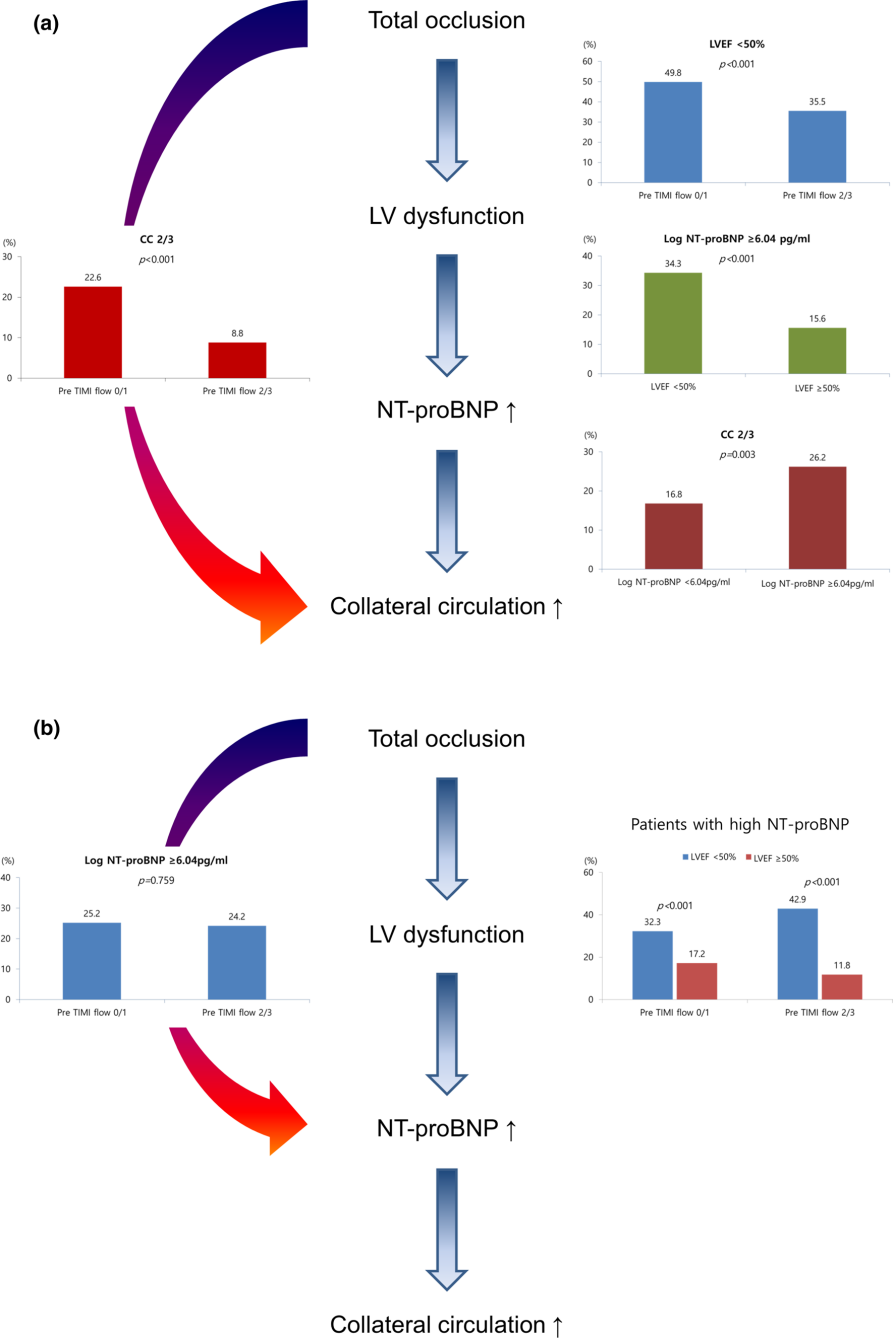

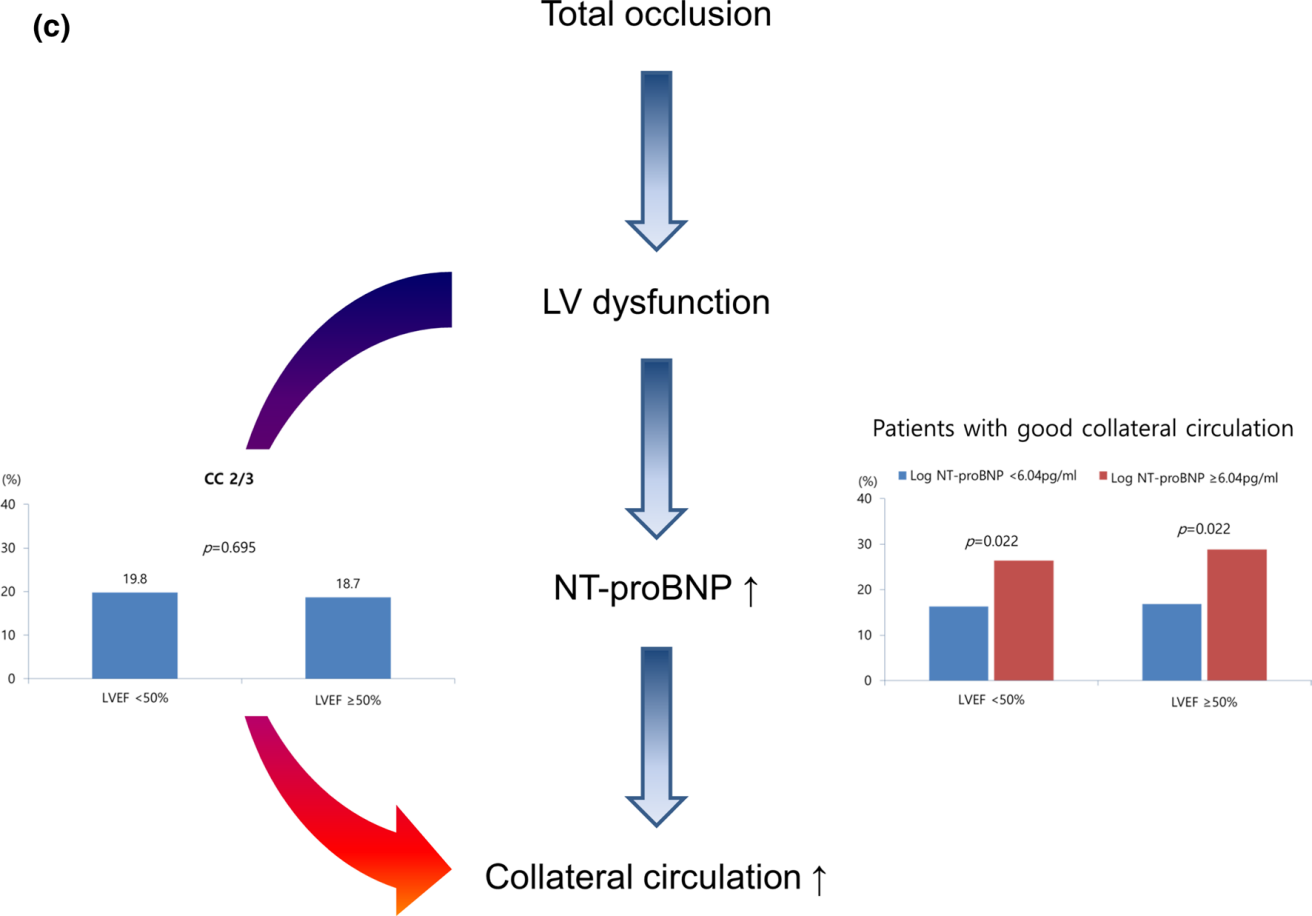
**a** Plausible mechanism of the development collateral circulation. Left, relationship between pre-TIMI flow and collateral circulation. Right upper, relationship between pre-TIMI flow and LV dysfunction. Right middle, relationship between LV dysfunction and log NT-proBNP. Right lower, relationship between log NT-proBNP and collateral circulation. **b** Plausible mechanism of the development collateral circulation. Left, relationship between pre-TIMI flow and log NT-proBNP. Right, relationship between LV dysfunction and NT-proBNP, regardless of pre-TIMI flow. CC: collateral circulation, LVEF: left ventricular ejection fraction. **c** Plausible mechanism of the development collateral circulation. Left, relationship between LVEF and collateral circulation. Right, relationship between log NT-proBNP and collateral circulation regardless of LV dysfunction. *CC* collateral circulation, *LVEF* left ventricular ejection fraction

Good CC was greater in patients with a pre-TIMI flow of 0 or 1 (22.6% versus 8.8%, *p* = 0.001) (Fig. 6a). LV dysfunction (LVEF < 50%) was greater in patients with a pre-TIMI flow of 0 or 1 (49.8% versus 35.5%, *p* < 0.001). High NT-proBNP levels were more common in patients with LV dysfunction (34.3% versus 15.6%, *p* < 0.001). Good CC was greater in patients with high NT-proBNP levels (16.8% versus 26.2%, *p* = 0.003).

There was no significant difference in patients with a pre-TIMI flow of 0 or 1 between those with log NT-proBNP levels ≥ 6.04 pg/ml and those with log NT-proBNP levels < 6.04 pg/ml (25.2% versus 24.2%, *p* = 0.759) (Fig. 6b). High NT-proBNP was significantly greater in patients with LV dysfunction regardless of pre-TIMI flow.

There was no significant difference between patients with good CC among those with and without LV dysfunction (19.8% versus 18.7%, *p* = 0.695) (Fig. 6c). Good CC was significantly greater in patients with high NT-proBNP levels regardless of LV function.

## Discussion

The principle findings of this study were as follows. First, an elevated level NT-proBNP was associated with collateral development in patients undergoing primary PCI after STEMI. Second, an elevated level of serum NT-proBNP was an independent predictor of good CC. Third, a pre-TIMI flow of 0 or 1 was associated with good CC, but it was not associated with NT-proBNP. Fourth, LV function was associated with NT-proBNP but not with good CC. Therefore, the relationship between NT-proBNP and CC was not influenced by pre-TIMI flow and LV function.

To the best of our knowledge, this is the first study to demonstrate the association between NT-proBNP and the formation of collateral arteries in patients who underwent primary PCI after STEMI. Previous studies have shown that the extent of collateral circulation is affected by age, sex, presence of pre-TIMI flow grade of 0 or 1, anterior wall AMI, multivessel disease, preinfarction angina, and time from symptom onset to cardiac catheterization [14–17]. A recent study demonstrated the association between BNP level and the development of CCs in chronic stable angina [18]. Our study adds novel insight to the previous finding that the NT-proBNP level is related to the development of CC, even after adjusting confounding variables and is an independent predictor of collateral formation in STEMI.

Several studies have demonstrated that BNP is overexpressed in the ischemic myocardium [19, 20]. The time course of the plasma level of BNP in patients with AMI can be divided into monophasic and biphasic patterns [21]. The plasma levels and the presence or absence of the second peak of plasma BNP in the subacute phase appear to reflect the degree of LV dysfunction and infarct size. However, an early increase within 4–8 h after the onset of symptoms is not solely related to the degree of LV dysfunction and infarct size. In the present study, the serum NT-proBNP levels were significantly higher in patients with good CC. However, there was no significant difference in LVEF between patients with and without good CC. This suggests that an early increase of NT-proBNP within 4–8 h after the onset of symptoms is associated with collateral formation. BNP in the early phase of AMI is one of the acute-phase reactants that are released in response to myocardial necrosis, local mechanical stress, or both on ventricular myocytes, even when global hemodynamic parameters are within the normal range [21, 22].

Although we found that the circulating NT-proBNP levels in early phase of AMI correlated with the degree of collateral formation, the causative mechanisms remain unclear. In patients with STEMI, myocardial ischemia is a potent trigger for collateral formation, and the severity of myocardial ischemia is positively correlated with collateral formation. In the present study, CC development was more common in patients with a pre-TIMI flow of 0 or 1 than those with a pre-TIMI flow of 2 or 3. Therefore, we hypothesized that total occlusion in STEMI results in LV dysfunction, which causes myocardial wall stress, and that NT-proBNP is released from the ventricular myocardium. This increase in NT-proBNP could lead to the development of CC. The results of our study support our hypothesis as follows. LV dysfunction was significantly more prevalent in patients with a pre-TIMI flow of 0 or 1. NT-proBNP was released more in patients with LV dysfunction. Finally, this release of NT-proBNP was associated with the development of CC. Interestingly, the release of NT-proBNP was not associated with pre-TIMI flow, but it was associated with LV dysfunction. Moreover, LV dysfunction was not associated with the development of CC, but it was associated with the release of NT-proBNP. As a result, the circulating NT-proBNP level in the early phase of AMI is independently associated with collateral formation.

Although we hypothesized that an increase of NT-proBNP could lead to the development of CC, it is uncertain whether this association is causal or a mere epiphenomenon. Recent animal studies revealed that BNP is a strong stimulator for angiogenesis in ischemic conditions. A previous study reported that transgenic mice overexpressing BNP showed improved reendothelialization after induced ischemia [23]. Recently, BNP has been reported to act as a potent vasculogenic agent by enhancing the number, proliferation, adhesion, and migration of endothelial progenitor cells through paracrine activation of the receptor guanylyl cyclase-A [9, 10]. This suggests that BNP plays a central role in endothelial homeostasis and acts as a regulator of vascular tone and endothelial regeneration [24–27]. The results of our study are consistent with those of previous animal studies that showed that the CC grade increases as the serum level of NT-proBNP rises. Therefore, we assumed that NT-proBNP has provasculogenic properties to promote collateral formation in post-MI patients. Although there is no management strategy based on early elevated BNP levels for patients with STEMI at this time, NT-proBNP could be useful for monitoring the effectiveness of angiogenic therapy and identifying individuals in need of such treatment in the near future.

In the present study, we hypothesized that serum NT-proBNP levels would be elevated for angiogenesis in the acute stage in patients with STEMI because of severe myocardial ischemia. Accordingly, CC develops as the serum NT-proBNP level rises. In the conventional concept, good CC may preserve LVEF leading to low BNP (Supplementary Fig. 1A). However, BNP is also strongly correlated with the severity of myocardial ischemia, and an elevated level of BNP is, therefore, positively associated with good CC (Supplementary Fig. 1B). Therefore, in the novel concept, BNP is a strong stimulator for angiogenesis reflecting myocardial ischemia, and as a result, good CC would be developed immediately after STEMI.

Our research has several limitations that should be considered. First, because this was an observational study, we cannot completely exclude the possibility of residual confounding factors, although we attempted to control for selection bias with multivariate statistical methods. Therefore, our results should only be regarded as hypothesis generating. Second, we assessed coronary collateralization by Rentrop’s score instead of the collateral flow index. However, Rentrop’s score was used extensively in previous studies investigating the collateral development in humans and was shown to be a well-documented and reproducible method to assess coronary collateral vessels. Therefore, these limitations should not undermine the strength of this study.

In conclusion, the serum level of NT-proBNP was increased markedly in patients with good CC. Although the precise mechanisms by which NT-proBNP is synthesized and released remain to be elucidated, the serum level of NT-proBNP appears to reflect the degree of collateral formation in the early phase of AMI.

## Supplementary Information

Below is the link to the electronic supplementary material.

**Supplementary Figure**. Conventional concept (A) versus novel concept (B) regarding BNP and collateral formation immediately after STEMI. (TIF 83 kb)
