# Supplementary figures and images for: N-terminal pro-brain natriuretic peptide and coronary collateral formation in patients undergoing primary percutaneous coronary intervention
Source: Heart Vessels. 2021 May 28;36(12):1775–83. doi: 10.1007/s00380-021-01866-3 (PMC8556172; doi:10.1007/s00380-021-01866-3)

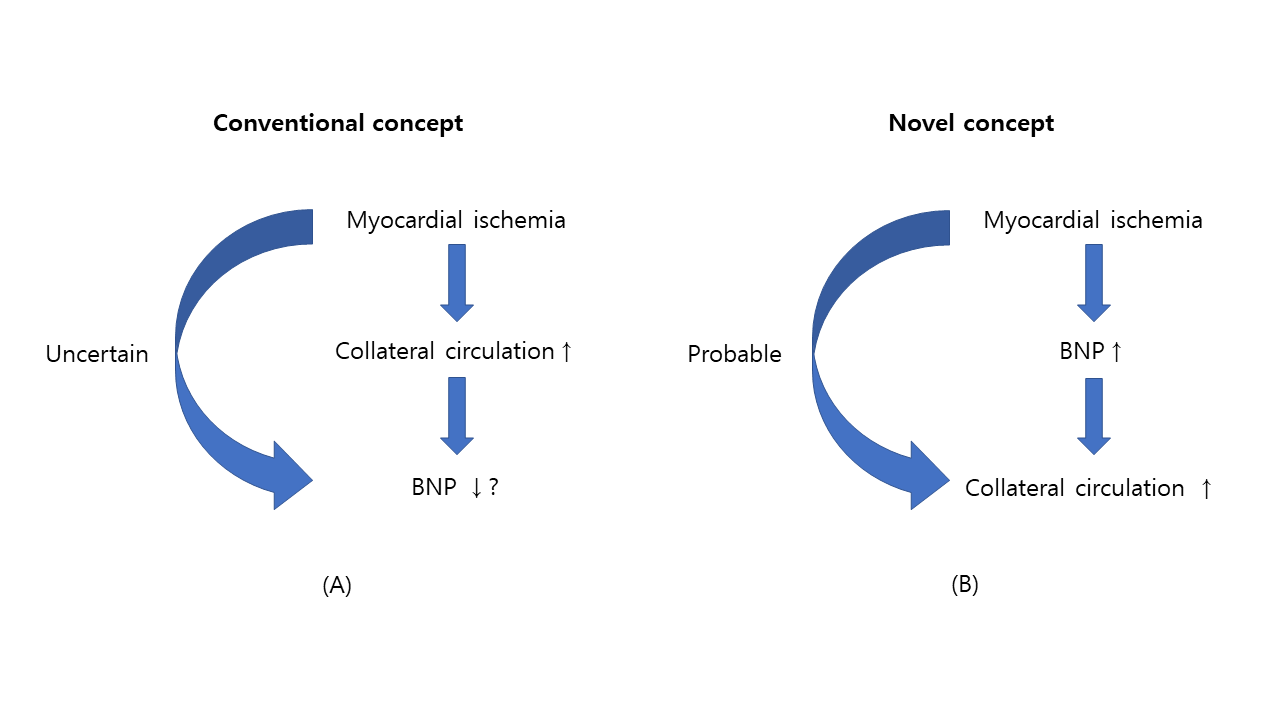

Supplement: Supplementary file 1 — Supplementary Figure. Conventional concept (A) versus novel concept (B) regarding BNP and collateral formation immediately after STEMI. (TIF 83 kb) [file 380_2021_1866_MOESM1_ESM.tif]
